# Successful treatment with tislelizumab plus chemotherapy for SMARCA4-deficient undifferentiated tumor: a case report

**DOI:** 10.3389/fimmu.2024.1371379

**Published:** 2024-05-31

**Authors:** Wen Dong, Anli Dai, Zhijun Wu, Jiangtao Wang, Tao Wu, Yangfeng Du, Wei Tian, Jiang Zheng, Yan Zhang, Hongming Wang, Juan Cai, Susu Dong, Yan Zhou, Siyan Li, Zemin Xiao

**Affiliations:** ^1^ Department of Oncology, Changde Hospital, Xiangya School of Medicine, Central South University (The First People’s Hospital of Changde City), Changsa, China; ^2^ Department of Pathology, Changde Hospital, Xiangya School of Medicine, Central South University (The First People’s Hospital of Changde City), Changsa, China; ^3^ Department of Respiratory, Changde Hospital, Xiangya School of Medicine, Central South University (The First People’s Hospital of Changde City), Changsa, China

**Keywords:** SMARCA4-dUT, tislelizumab, immune therapy, Anti-PD-1 antibody, chemotherapy

## Abstract

SMARCA4-deficient undifferentiated tumor (SMARCA4-dUT) is a devastating subtype of thoracic tumor with SMARCA4 inactivation and is characterized by rapid progression, poor prognosis, and high risk of postoperative recurrence. However, effective treatments for SMARCA4-dUT are lacking. Herein, we describe a patient with SMARCA4-dUT who exhibited an impressive response to the anti-programmed cell death protein-1 (PD-1) antibody (tislelizumab) in combination with conventional chemotherapy (etoposide and cisplatin). To the best of our knowledge, this is the first case of SMARCA4-dUT treated with chemotherapy, comprising etoposide and cisplatin, combined with anti-PD-1 inhibitors. Immunotherapy combined with etoposide and cisplatin may be a promising strategy to treat SMARCA4-dUT.

## 1 Introduction

The new 2021 World Health Organization (1) Classification of Tumors of the Lung, Pleura, Thymus, and Heart has renamed the entity previously described as SMARCA4-deficient thoracic sarcoma (SMARCA4-dTS) to SMARCA4-deficient undifferentiated tumor (SMARCA4-dUT) (2). SMARCA4-dUT is a devastating subtype of lung cancer with a poor prognosis and a median survival time of < 6 months, despite surgical intervention (3).

The optimal treatment for SMARCA4-dUT has not been determined due to a lack of evidence. Immunotherapy seems to be a feasible strategy for SMARCA4-dUT. Potential treatment regimens include mono pembrolizumab (4, 5), mono nivolumab (6), mono tislelizumab (7), ipilimumab combined with pembrolizumab (8), and ABCP (atezolizumab in combination with bevacizumab, paclitaxel, and carboplatin) (9, 10). Although chemotherapy with etoposide and cisplatin (EP) is widely employed for thoracic malignancies, especially to treat tumors with poor prognosis, such as small cell lung cancer, neuroendocrine carcinoma, and mediastinal vitelline cyst tumors, the efficacy of EP regimen for SMARCA4-dUT remains unclear. Herein, we present the first case of successful treatment of SMARCA4-dUT with tislelizumab, an anti-programmed cell death protein-1 (PD-1) inhibitor, in combination with conventional cytotoxic agents (such as EP).

## 2 Case presentation

**Figure 1:**
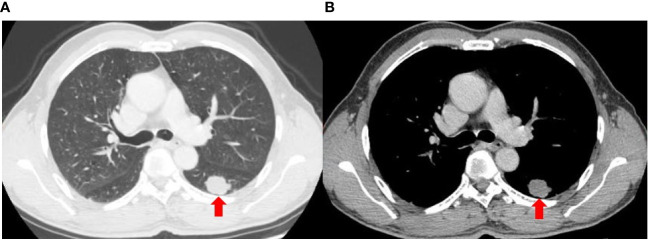
Axial computed tomography images of the lung **(A)** and mediastinal **(B)** window show a lobular soft tissue mass in the left lower lobe.

**Figure 2:**
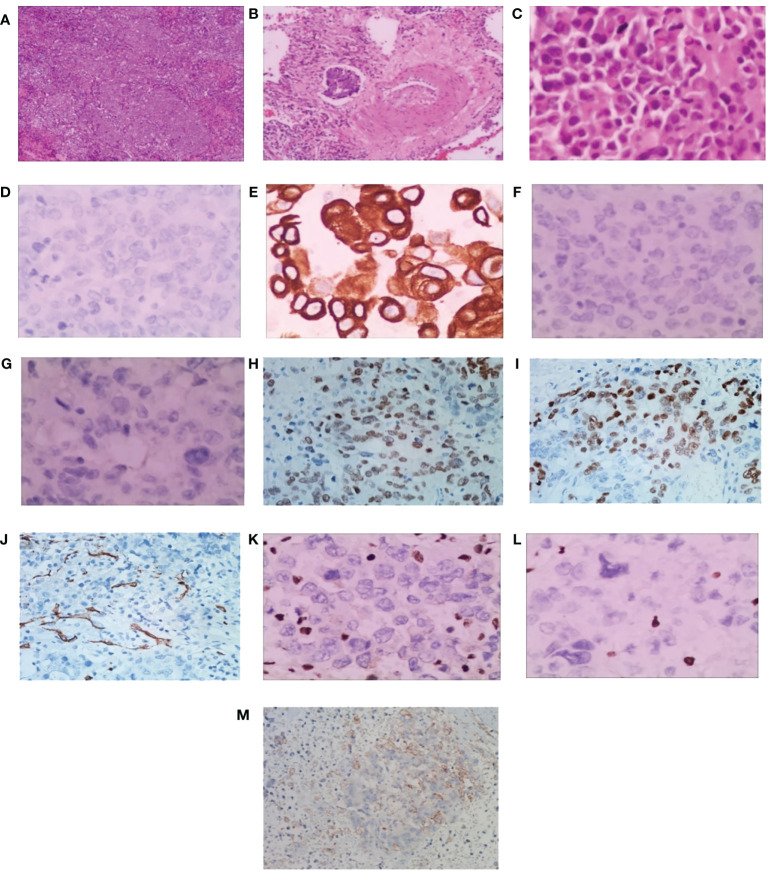
Pathological findings of surgical specimens. **(A)** Low-power view showing diffuse sheets and nests of slightly discohesive cells (H&E). Scale bar = 500 μm. **(B)** Vascular cancer thrombus (H&E). Scale bar = 200 μm. **(C)** Big round tumor cells, abundant eosinophilic cytoplasm with eccentric nuclei, and a high mitotic rate (H&E). Scale bar = 50 μm. **(D)** panCK, **(E)** CK7, **(F)** TTF-1 and **(G)** NUT showing tumor cell nonreactivity. **(H)** SALL-4 showing tumor cell reactivity, and **(I)** SOX2 showing focal tumor cell reactivity. The tumor cells display nonreactivity for **(J)** CD34, and vessels show reactivity for CD34. Both **(K)** SMARCA4 and **(L)** SMARCA2 are completely lost. Scale bar = 50 μm. **(M)** The programmed death ligand-1 tumor proportion score (PD-L1 TPS) is 1%, and the combined positive score (PD-L1 CPS) is 5. Scale bar = 10 μm. H&E, hematoxylin-eosin.

A 56-year-old heavy smoker (Brinkman index: 600) was referred to The First People’s Hospital of Changde City with sudden onset coughing lasting approximately three weeks. The patient expressed fear regarding inadequate tumor control and the potential for a life-threatening illness. The patient had no previous history of tumors, and there were no issues with the axillary lymph nodes at the time of surgery. Apart from the aforementioned symptoms, physical examination and patient history afforded unremarkable findings. No risk factors or familial background for neoplasms were identified. Previously, the patient underwent a treatment regimen comprising chemotherapy and immunotherapy, followed by maintenance immunotherapy. Computed tomography (CT) revealed a thoracic mass in the left lower lobe (**Figure 1**). Preoperative CT imaging revealed nodular thickening in the medial and combined branches of the left adrenal gland, with mild enhancement observed during the enhancement scan. Conversely, the right adrenal gland exhibited no obvious abnormalities. Additionally, cystic hypodense shadows were observed in both kidneys, with no significant enhancement noted during the enhancement scan. Moreover, no enlarged lymph nodes were detected in the retroperitoneum (**Supplementary Figure S1**). Preoperative magnetic resonance imaging (MRI) scan of the head showed no significant abnormalities in the morphologic signals of the plasma membrane, brain cell system, and brain surface sulci. The midline was centered and no significant abnormalities were seen in the bones and soft tissues (**Supplementary Figure S2**). The patient subsequently underwent left lower lung resection, and the resected tumor mainly comprised undifferentiated carcinoma of large round cells. Immunohistochemical staining results were as follows (**Figure 2**): panCK (AE1/AE3), CK7 (OV-TL12/30), TTF-1 (SP141), and NUT (GTX34106) negative; SALL-4 (SP289) reactive; SOX2 (BP6123) focally reactive; tumor cells negative for CD34 (HK11190) and vessels reactive for CD34 (HK11190); SMARCA4 (EPNCIR111A) and SMARCA2 (HPA029981) completely lost. The patient was finally diagnosed with SMARCA4-deficient undifferentiated tumor (SMARCA4-dUT) with no targetable driver mutations. The programmed death ligand-1 tumor proportion score (PD-L1 TPS) was 1%, with a combined positive score (PD-L1 CPS) of 5. The pathological stage was T1bN0M0 IB (according to AJCC Cancer Staging Manual, Version 8).

**Figure 3:**
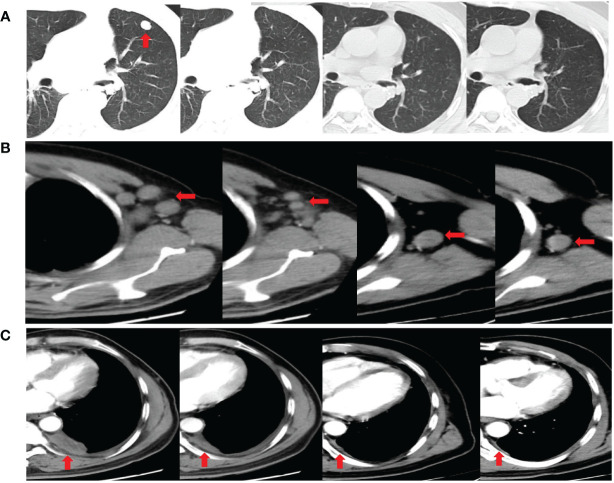
Chest computed tomography images showing the disappearance of the left lung metastasis **(A)**, the shrinkage of the left axillary lymph nodes **(B)**, and the shrinkage of the left pleural dissemination **(C)** after completing six cycles of tislelizumab plus etoposide and cisplatin induction therapy. Chest computed tomography images demonstrating a durable response after completing ten doses and fourteen doses of tislelizumab maintenance phase (Partial axillary lymph nodes disappeared after ten doses of tislelizumab).

**Figure 4:**
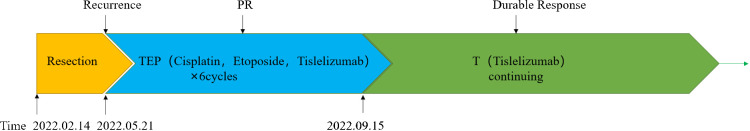
Time since treatment initiation.

Three months after surgery, the patient rapidly developed disease recurrence, including left lung, left mediastinal lymph nodes, left axillary lymph nodes, and left pleural metastases. Symptoms of compression in the axillary lymph nodes included discomfort and a sensation of a foreign body. The patient was able to palpate the lymph nodes, which were hard, approximately 2 cm × 2 cm in size, with unclear borders, poor mobility, and no tenderness. The postoperative MRI scan of the head revealed speckled long T1T2 signal shadows surrounding the ventricles bilaterally, with a high signal observed on TIR. No evident abnormal enhancement foci were detected upon enhancement. The ventricular morphology signal appeared intact, with a centered midline, and no apparent abnormalities were noted in the sulcal fissure. Additionally, mucosal thickening was observed in the paranasal sinuses. Moreover, a mound-like short T1 signal was observed in the subcutis of the left parietal region (**Supplementary Figure S2**). Six cycles of tislelizumab plus EP induction therapy were administered as first-line chemotherapy, with cisplatin (40 mg/m^2^ on days 1–3), etoposide (100 mg/m^2^, days 1–3), and the immune checkpoint inhibitor (ICI) tislelizumab (200 mg, day 1), and then switched to the maintenance phase (continued tislelizumab 200 mg, day 1) without any special side effects (only mild nausea). Chest CT images (**Figure 3**) were obtained after completing six courses of tislelizumab plus EP, revealing the disappearance of the left lung metastasis, shrinkage of all lymph nodes, and left pleural dissemination (best overall response: partial response, according to the RECIST version 1.1). Postoperative CT imaging of the lungs showed that the bronchial tubes in the dorsal segment of the lower lobe of the left lung were not shown, and a metallic dense shadow was seen, which was a postoperative change; the vascular bronchial bundles of the two lungs were increased, thickened and fuzzy, and the translucency of both lungs was increased, and cystic translucent shadows of varying sizes were seen; a nodular shadow of about 12 mm × 11 mm was seen in the upper lingual segment, with clear borders and mild enhancement; a mass-like hyperdense shadow of about 28 mm × 14 mm was seen in the posterior segment of the upper lobe of the left lung, and it was moderately unevenly intensified. In the remaining lungs, there were scattered small nodules and flocculent shadows, and enlarged lymph nodes were seen in the mediastinum and the left hilar, with blurred borders and uneven enhancement, and irregular fluid density shadows were seen in the left thoracic cavity and nodular thickening of the pleura, with uneven enhancement. Multiple enlarged lymph nodes were seen in the left axilla, partially fused and with blurred borders. The liver parenchyma is shown to be hypodense. Nodular non-enhancing foci are seen in both kidneys. Thoracic spine bone is unevenly hypodense (**Supplementary Figure S3**). The patient continued treatment for 9 months without disease progression (**Figure 4**).

## 3 Discussion

SMARCA4-dUT was first defined in the 5th edition of the WHO Classification of Thoracic Tumors, published in 2021 (1). SMARCA4-dUT was initially defined as SMARCA4-dTS, originally discovered in 2015 by Le Loarer et al. (11) by RNA sequencing of unclassified sarcomas, and subsequently classified as a new type of undifferentiated lung malignancy of pulmonary epithelial origin. Thoracic SMARCA4-dUT exhibits consistent immunohistochemical loss of both SMARCA4 and SMARCA2 (12). In addition, this tumor frequently displays the robust expression of one or more stem cell markers, such as SOX2, CD34, and SALL4, which can facilitate the differential diagnosis of SMARCA4-deficient non-small cell lung cancer (NSCLC) (SMARCA4-dNSCLC), typically negative for these markers (13, 14). SMARCA4-dNSCLC usually expresses more diffuse and stronger keratin than thoracic SMARCA4-dUT and is often negative for thyroid transcription factor (TTF)-1 (15). Morphologic features of SMARCA4-dUT can be either undifferentiated small-cell or large-cell malignancies (16, 17). All areas of the tumor are undifferentiated with slightly discohesive cells arranged in sheets and nests, abundant geographic necrosis. Cytologic features included many areas with rhabdoid cells and a high mitotic rate. In this case, the tumor is mainly comprised of undifferentiated carcinoma of large round cells, cytologic features (undifferentiated tumor cells with slightly discohesive cells arranged in sheets and nests, abundant geographic necrosis), and immunohistochemical staining results (complete loss of both SMARCA4 and SMARCA2, reactivity of SALL-4) matching histopathology characteristics of SMARCA4-dUT.

SMARCA4-dUT is common in heavy male smokers and has a poor prognosis, with early postoperative recurrence in operable patients (10). Typically, patients present with large, infiltrative, and compressive thoracic masses frequently associated with necrotic lymphadenopathy (13, 14). Although mediastinal involvement may be prominent, most cases present with focal parenchymal continuity, often with substantial emphysema. Metastatic involvement is common, involving the lymph nodes, bones, lungs, brain, and adrenal glands, a pattern notably similar to that observed in NSCLC (18). SMARCA4-dUT also can be central or peripheral lung lesions, with involvement of the parietal pleura and chest wall. In general, the clinical presentation and radiological features of SMARCA4-dUT are nonspecific (19). In this case, the patient presented with a peripheral lung lesion and involvement of lymph nodes.

Thoracic SMARCA4-dUT is a new type of devastating neoplasm, and most cases of SMARCA4-dUT exhibit an advanced stage at presentation with considerably poor survival outcomes (20). SMARCA4-dUT of the thorax is a rare but aggressive neoplasm primarily occurring in heavy-smoking adults aged 30–59 years (21). The median overall survival (OS) ranges from five to seven months, with a 2-year OS of only 12.5% with traditional therapies (11, 13, 22, 23). SMARCA4-dUT carries a high risk of recurrence after surgical resection, even among stage I (23). In this case, the patient had a relapse (intrathoracic recurrence and axillary lymph node metastasis) less than 10 weeks after surgery.

Common mutations in SMARCA4-dUT include *TP53*, *KRAS*, *STK11*, *KEAP1*, *ARID1A*, and *NF1* (24). Currently, guidelines for treating SMARCA4-dUT are yet to be established. Few reports have explored the feasibility of ICI combined with chemotherapy for thoracic SMARCA4-dUT, demonstrating that immunotherapy might be a promising strategy for treating thoracic SMARCA4-dUT (4–10). The marked response to ICI therapy may be mediated via dysfunction of polybromo‐ and Brahma‐related gene 1 (BRG1)‐associated factor (PBAF) associated with the loss of SMACA4 (24). SMARCA4 is a subunit of the switch/sucrose non-fermentable (SWI/SNF) chromatin-remodeling complex. Reisman et al. (25, 26) have demonstrated that loss of SMARCA4 can occasionally be observed in otherwise conventional NSCLC and is associated with a markedly aggressive clinical course. Loss of PBAF function may induce the upregulated expression of interferon (IFN)-γ-responsive genes and the secretion of T cell chemoattractants to recruit effector T cells to tumors (24). Therefore, dysfunction of such chromatin-remodeling complexes can lead to increased efficacy of anti-PD-1 blockade against malignancies. Accumulating evidence has revealed that the clinical benefits of ICI can be associated with the loss of function of PBAF complexes in a subset of neoplasms (27).

ICI is associated with largely improved survival among SMARCA4-dUT (28). There have been few reports on the effectiveness of ICI in SMARCA4-dUT, which might be a new promising therapy option. Among immunotherapy-based strategies, mono-immunotherapies [such as pembrolizumab (4), tislelizumab (7) and nivolumab (29)], combined immunotherapy [Ipilimumab and pembrolizumab (8)], and combined immunotherapy with chemotherapy (ABCP) (9, 10) have been explored. ABCP is the most popular of these therapies owing to its durable response. According to reported cases, ABCP, when used as the first-line therapy, could provide progression-free survival (PFS) ranging from 6 to >17 months; however, the OS was only several months without immunotherapy (30). Chemotherapy with EP can induce sufficient antitumor activity with acceptable toxicity in thoracic cancer, especially in tumors with a poor prognosis, including small cell lung cancers, mediastinum vitelline cyst tumors (31), and neuroendocrine carcinomas (32). SMARCA4-dUT is a novel thoracic malignancy with poor survival outcomes. Herein, we attempted to treat a patient with SMARCA4-dUT using tislelizumab (an anti-PD-1 antibody) in combination with EP. The patient experienced a durable response (more than 9 months). TEP (tislelizumab in combination with etoposide and cisplatin) dramatically suppressed the growth of malignancies. The objective response rate (ORR) for SMARCA4-dUT patients treated with ICI was only 50% (23). The favorable ABCP can provide PFS ranging from 6 to > 17 months, while other immunotherapies can provide 5 to >14 months (4–10). Without immunotherapy, the median OS for SMARCA4-dUT is only 5.2 months (14). In our case, a CT scan confirmed an impressive partial response, and PFS is more than 9 months with TEP. The > 9 months PFS is considerably excellent compared with other immunotherapies, including ABCP. The prognosis for SMARCA4-dUT cannot always be reliably predicted based solely on PD-L1 expression. Even cases with negative or low PD-L1 expression might exhibit a lasting response to immune therapies. At least 3 reported cases with negative PD-L1 expression exhibited more than 10 months of PFS. TEP also provided > 9 months PFS for the patient with PD-L1 TPS 1% in this case. Case reports of SMARCA4-dUT treated with ICI are summarized in **Table 1**. Although there is still a lack of evidence of large-scale clinical trials for chemotherapy combined with immunotherapy in SMARCA4-dUT, tislelizumab plus EP is likely to become one of the most popular strategies for SMARCA4-dUT for economic reasons. EP is a widely used chemotherapy with a lower cost than ABCP, and the cost of tislelizumab plus EP therapy is less than one-tenth that of ABCP therapy in China (33). Furthermore, bevacizumab is unsuitable for patients with symptoms of hemoptysis, a markedly common symptom in patients with thoracic malignancies (34). Therefore, tislelizumab plus EP is a promising strategy for treating SMARCA4-dUT.

**Table 1:** Case reports of SMARCA4-UT treated with immunotherapy.

No.	Author	Age (Y)/gender	Smoking status	Lesion location	TMN	PD-L1 TPS (%)	TMB (/Mb)	Mutation	Treatment	Outcome (follow-up)
1	Kawachi et al. (7)	73/F	53 pack-years	center upper lobe, mediastinal lymphadenopathy and osteolytic metastasis in the fifth cervical vertebra	IVB	40	11	SMARCA4 (c.1119- 1G>T), TP53, SPTA1, CHD2	ABCP	Progressed after 17 months of PR to 2021
2	Kawachi et al. (7)	59/M	39 pack-years	center lower lobe, an oropharyngeal mass, center adrenal gland mass, center cervical lymphadenopathy, center hilar lymphadenopathy and multiple abdominal lymphadenopathies	IVB	0	11.8	SMARCA4 (K755Nfs), TP53, TSC2	ABCP	Progressed after 10 months of PR to 2021
3	Kawachi et al. (7)	64/F	44 pack-years	center lower lobe, an oropharyngeal mass, center adrenal gland mass, center cervical lymphadenopathy, center hilar lymphadenopathy and multiple abdominal lymphadenopathies	IVB	80	14.9	SMARCA4 (H103Mfs), TP53, SPTA1, TSC2, KEAP1	ABCP	Progressed after 7 cycles of PR and 2 months maintenance therapy to 2021
4	Naito et al. (21)	43/M	Current smoker	center upper lobe, multiple lung metastases	IVA	0	Unknow	Unknow	nivolumab	14 months PR to January 2019
5	Kunimasa et al. (8)	51/M	22.5 pack-years	right upper lobe with soft tissue and vertebral invasion	IVA	0	10.2	SMARCA4 (L1161fs), TP53 (V157L)	ABCP	9 months PR after surgery to August 2021
6	Utsumi et al. (13)	72/M	80 pack-years	right pleural thickening, and pleural effusion	IVA	Unknow	Unknow	Unknow	ACP	SD and 7 months to death
7	Anžič N et al. (6)	41/M	Current smoker	Upper anterior mediastinum encompassing infracarinal and hilar lymph nodes	IV	100	TMB-H	CDKN2A (p.T18fs), SMARCA4 (p.K1334fs)	Pembrolizumab + ipilimumab	25 months to death
8	Henon et al. (2)	58/F	Unknown	Mediastinal, hilar and paratracheal involvement, along with pleural and peritoneal metastases	IV	0	TMB-L	Unknown	Pembrolizumab	11 months PR to March 2019
9	Shi L et al. (5)	50/M	36 pack-years	center upper lobe of the lung involving the pulmonary artery, pleural and pericardial effusion	IVA	90	23.93	SMARCA4 c.797C>T (p. Ser266*), KEAP1, TP53, and TERT	Tislelizumab	7 cycles of PR to August 2022
10	Takada et al. (3)	69/F	Unknow	center mediastinal tumor, peritoneal and retroperitoneal dissemination and multiple cutaneous metastases	IVC	60	Unknown	Unknown	Pembrolizumab	8 cycles of PR to October 2019
11	Iijima et al. (4)	76/M	49 pack-years	lymphadenopathy at the right neck and mediastinum, a large anterior mediastinal mass and small nodules in the right upper lung	IV	0	Unknown	negative results for EGFR, ALK, ROS-1 and BRAF	nivolumab	22 months PR to August 2019

ABCP, atezolizumab, bevacizumab, paclitaxel, and carboplatins.

ACP, atezolizumab, nab-paclitaxel, and carboplatins.

SD, stable disease.

Given the limitations of single case reports, the efficacy of anti-PD-1 antibodies combined with EP chemotherapy needs to be further validated in a larger patient cohort with SMARCA4-dUT.

## Data availability statement

The original contributions presented in the study are included in the article/**Supplementary Material**. Further inquiries can be directed to the corresponding author.

## Ethics statement

The study was reviewed and approved by the Institutional Review Board of the First People’s Hospital of Changde City, China. The patient provided written informed consent for study participation. Written informed consent was obtained from the patient/participant for the publication of this case report.

## Author contributions

WD: Writing – original draft. AD: Writing – original draft. ZW: Writing – review & editing. JW: Writing – review & editing. TW: Writing – review & editing. YD: Writing – review & editing. WT: Writing – review & editing. JZ: Writing – review & editing. YZha: Writing – review & editing. HW: Writing – review & editing. JC: Writing – review & editing. SD: Writing – review & editing. YZho: Writing – review & editing. SL: Writing – review & editing. ZX: Writing – original draft, Writing – review & editing.
