# Supplementary material for: Successful treatment with tislelizumab plus chemotherapy for SMARCA4-deficient undifferentiated tumor: a case report
Source: Front Immunol. 2024 May 31;15:1371379. doi: 10.3389/fimmu.2024.1371379 (PMC11176515; doi:10.3389/fimmu.2024.1371379)
Supplement: Supplementary file 1 [file DataSheet_1.pdf]

-Supporting Information-

Successful Treatment with Tislelizumab Plus Chemotherapy for SMARCA4-deficient Undifferentiated Tumor: A Case Report

Wen Dong[1], Anli Dai[1], Zhijun Wu[1], Jiangtao Wang[2], Tao Wu[1], Yangfeng Du[1], Wei Tian[1], Jiang Zheng[1], Yan Zhang[1], Hongming Wang[1], Juan Cai[1], Susu Dong[3], Yan Zhou[1], Siyan Li[1], Zemin Xiao[1]*

[1] Department of Oncology, Changde Hospital, Xiangya School of Medicine, Central South University (The First People's Hospital of Changde City), China

[2] Department of Pathology, Changde Hospital, Xiangya School of Medicine, Central South University (The First People's Hospital of Changde City), China

[3] Department of Respiratory, Changde Hospital, Xiangya School of Medicine, Central South University (The First People's Hospital of Changde City), China

*** Corresponding author:** Zemin Xiao, **Email address:** xiaozemindoc@126.com

Figure S1

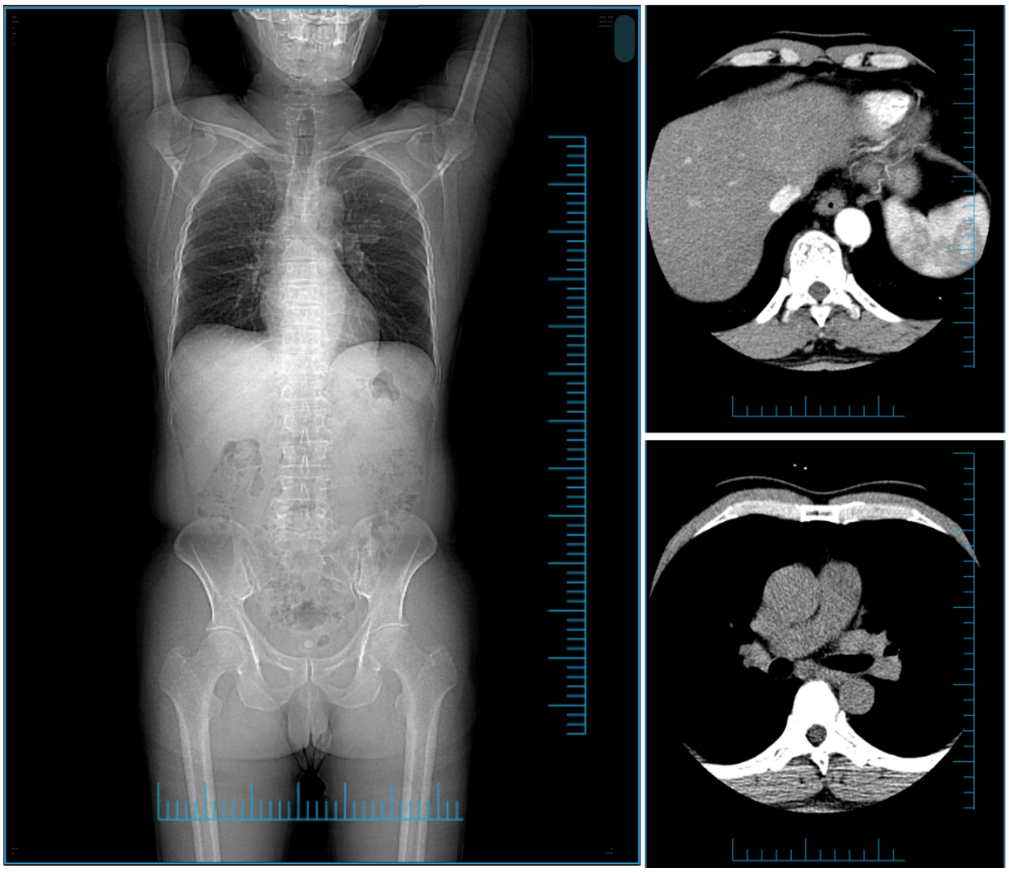

Figure S1. Preoperative CT imaging of adrenal glands. Nodular thickening of the medial and combined branches of the left adrenal gland with mild enhancement on enhancement scan. The right adrenal gland showed no obvious abnormality. Cystic low-density shadows were seen in both kidneys, with no significant enhancement on enhancement. No enlarged lymph nodes were seen in the retroperitoneum.

Figure S2

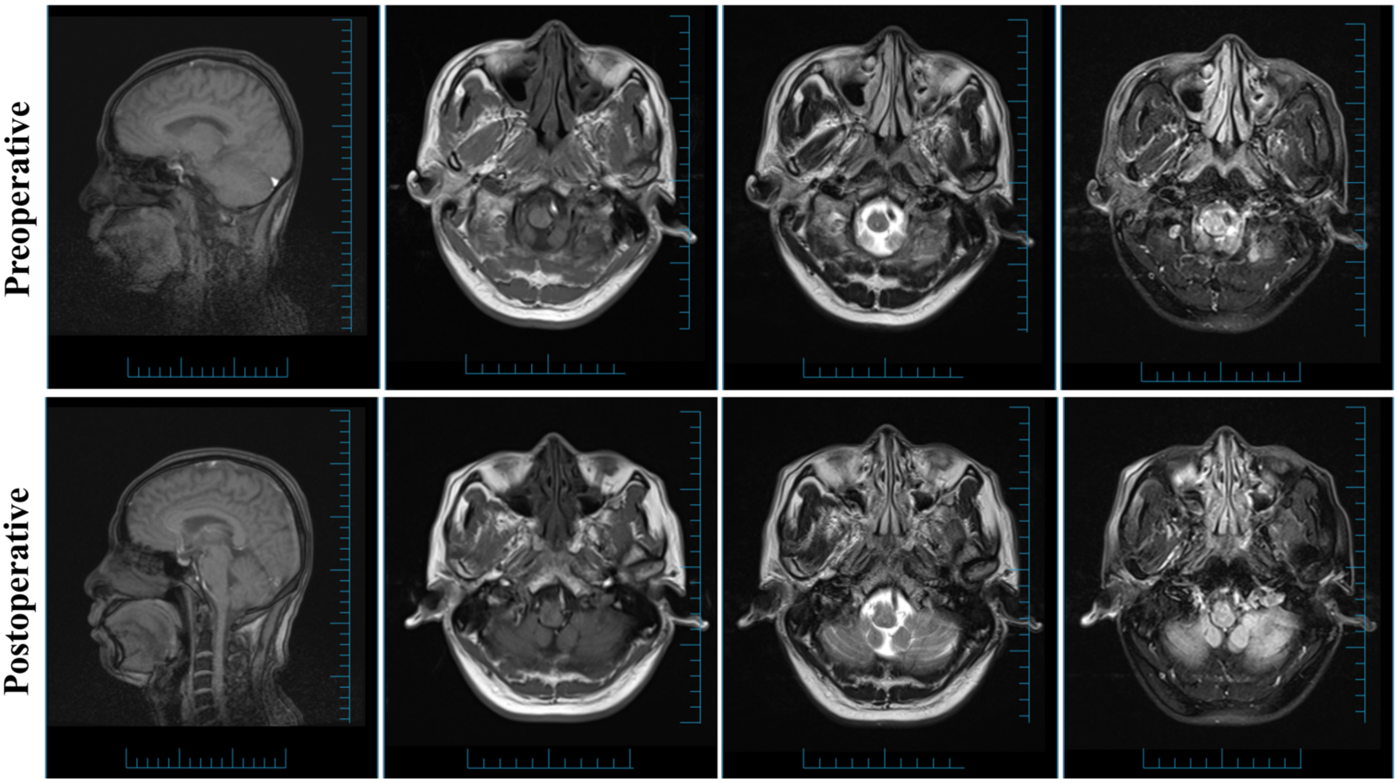

Figure S2. MRI scan of the head. Preoperatively: no significant abnormalities were seen in the morphologic signal of the cerebral plasmalemma, the cerebral cellar system, or the cerebral surface sulcus. The midline was centered, and there were no obvious abnormalities in the bone and soft tissues. The paranasal sinus mucosa was thickened. **Postoperatively:** speckled long T1T2 signal shadows were seen around the ventricles on both sides, TIR was a high signal, enhancement did not show obvious abnormal enhancement foci, ventricular morphology signal could be seen, the midline was centered, and the sulcal fissure had no obvious abnormality. Mucosal thickening of the paranasal sinuses. A mound-like short T1 signal was seen in the left parietal subcutis.

Figure S3

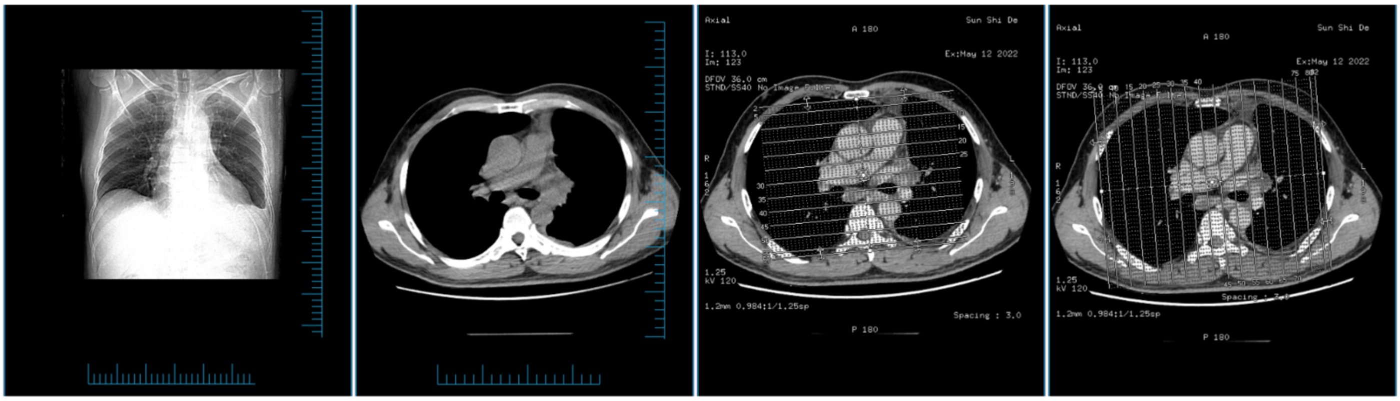

Figure S3. Postoperative CT imaging of the lungs. The bronchial tubes in the dorsal segment of the lower lobe of the left lung were not shown, and a metallic dense shadow was seen, which was a postoperative change; the vascular bronchial bundles of the two lungs were increased, thickened and fuzzy, and the translucency of both lungs was increased, and cystic translucent shadows of varying sizes were seen; a nodular shadow of about 12 mm × 11 mm was seen in the upper lingual segment, with clear borders and mild enhancement; a mass-like hyperdense shadow of about 28 mm × 14 mm was seen in the posterior segment of the upper lobe of the left lung, and it was moderately unevenly intensified. In the remaining lungs, there were scattered small nodules and flocculent shadows, and enlarged lymph nodes were seen in the mediastinum and the left hilar, with blurred borders and uneven enhancement, and irregular fluid density shadows were seen in the left thoracic cavity and nodular thickening of the pleura, with uneven enhancement. Multiple enlarged lymph nodes were seen in the left axilla, partially fused and with blurred borders. The liver parenchyma is shown to be hypodense. Nodular non-enhancing foci are seen in both kidneys. Thoracic spine bone is unevenly hypodense.
